# Monoinstitutional real world experience in management of Vinflunine as second line therapy for transitional cell carcinoma of the urothelium

**DOI:** 10.18632/oncotarget.24162

**Published:** 2018-01-11

**Authors:** Giovanni Schinzari, Ernesto Rossi, Francesco Pierconti, Giovanna Garufi, Santa Monterisi, Antonia Strippoli, Ettore D’Argento, Alessandra Cassano, Carlo Barone

**Affiliations:** ^1^ Department of Medical Oncology, Università Cattolica del Sacro Cuore, Rome, Italy; ^2^ Department of Pathology, Università Cattolica del Sacro Cuore, Rome, Italy

**Keywords:** vinflunine, transitional cell carcinoma of the urothelium, second line, toxicity

## Abstract

Vinflunine is the only cytotoxic agent tested as a second line therapy in transitional cell carcinoma of the urothelium in a phase III trial. It is not largely employed in clinical practice because of the high incidence of grade 3–4 toxicity. We evaluated efficacy and safety of Vinflunine at the dose of 280 mg/m^2^ every 3 weeks associated with primary prophylaxis with granulocyte growth factors and laxatives for patients progressed after platinum + Gemcitabine. Overall survival was 8.5 months, progression-free survival 4.33 months and response rate 25%, with disease control rate 57.2%. Grade III-IV neutropenia occurred in 10.7% of the patients, grade III-IV anemia and grade III thrombocytopenia in 10.7% and 7.2%, respectively. Among non haematological toxicity, grade I-II constipation was reported in 14.2% of the patients, without grade III-IV adverse events. No discontinuation for toxicity was observed. This study underlines that Vinfluinine at a dose of 280 mg/m^2^ associated with primary prophylaxis for neutropenia and constipation is effective and with a favorable toxicity profile.

## INTRODUCTION

Transitional cell carcinomas of the urothelium (TCCU) represent more than 90% of all cancers of the urinary tract, among which 90% are localized in the bladder. [1–2]. In Europe bladder cancer is the fifth diagnosed malignancy accounting for 4.7% of all human neoplasms [3].

Approximately half of the patients with muscle-invasive TCCU relapse after radical surgery, the majority of them with distant metastases. At the time of the diagnosis about 15% of patients have advanced disease [4]. Survival for untreated metastatic disease is no longer than six months [5]. The treatment of advanced stages of the disease is based on chemotherapy. Cisplatin-based regimens (Gemcitabine + Cisplatin: GC or Cisplatin + Methotrexate + Doxorubicin + Vinblastine: MVAC) are the cornerstone of first line treatment with a median survival of 13–15 months [6–7]. GC is generally preferred having a better toxicity profile [8]. GC and MVAC are also the standard of care in neoadjuvant/adjuvant setting. Prognosis is dismal at the time of progression after first line chemotherapy or recurrence after neoadjuvant/adjuvant treatment. When performance status is adequate and relapse occurs later than 12 months after neoadjuvant/adjuvant therapy, change of platinum based regimen could be considered [9]. Selected patients could be eligible to receive Paclitaxel plus Gemcitabine and Cisplatin [10]. In case of patients unfit for Cisplatin because of a poor performance status (up to 50% of patients) or impaired renal function, other options – e.g. alternative platinum agents (Oxaliplatin or Carboplatin) or the combination of Paclitaxel and Gemcitabine – have been proposed, although less effective [11–13]. The association with split dose Cisplatin and Gemcitabine could also be considered when the standard dose of Cisplatin cannot be administered [14]. Limited treatment options are available after an early relapse following platinum based neoadjuvant/adjuvant chemotherapy or after palliative first-line chemotherapy. The activity of agents, such as taxanes [15] and Pemetrexed [16], has been reported despite not being tested in randomized phase III trials.

Vinflunine is a microtubule inhibitor [17] approved by the European Medicine Agency (EMA) for use in TCCU (2009). Furthermore, it is recommended in ESMO guidelines as a second-line therapy for advanced bladder cancer [18] after first line chemotherapy for advanced disease or at time of recurrence after neoadjuvant/adjuvant treatment.

In the phase III trial [19, 20], Vinflunine was administered at a dose of 320 mg/m^2^ every 3 weeks for patients with performance status (PS) ECOG 0 and at a dose of 280 mg/m^2^ every 3 weeks for patients with PS ECOG 0 and a previous pelvic radiation or with PS ECOG 1. Median overall survival was 6.9 months for Vinflunine plus best supportive care versus 4.3 months for best supportive care solely. ORR was 8.6% for Vinflunine vs 0% for BSC, disease control rate 41.1% vs 24.8%, median PFS 3 vs 1.5 months, respectively.

An overall survival of 7–10 months was reported in other studies on efficacy and tolerability of Vinflunine conducted in Spain [21], Germany [22], France [23] and Italy [24].

In the phase III trial, half of the patients experienced grade 3–4 neutropenia. Other relevant grade 3–4 adverse events were: fatigue in 19.3% of the patients, constipation 16.1%, anemia 19.1% [19].

Despite being the only cytotoxic agent tested as a second line therapy in TCCU in phase III trials, Vinflunine is not largely employed in clinical practice due to a high incidence of grade 3–4 toxicity.

In order to evaluate the effectiveness and higher tolerability of the dose of 280 mg/m^2^ every 3 weeks associated with primary prophylaxis for neutropenia and constipation, we conducted this “real life” study including a group of patients with advanced urothelial carcinoma treated with this dose of Vinflunine in a single institution.

## RESULTS

The study included 28 patients with advanced metastatic TCCU treated with Vinflunine between January 2014 and December 2016. Patient characteristics are summarized in Table 1. Vinflunine was administered after first line platinum + Gemcitabine for advanced disease in 15 patients, whereas it was employed at the time of recurrence after neoadjuvant/adjuvant platinum + Gemcitabine in 13 patients.

**Table 1:** Patients’ characteristics

Patients	28
Median age	64.4 years
M/F	23/5
PS (ECOG)	
0	14
1	14
Platinum/Gem	
Neoadjuvant	10
Adjuvant	3
Metastatic	15
Median time from Neoadj/adj to relapse	9.7 months
Prior chemotherapy	
Cisplatin + Gemcitabine	20
Carboplatin + Gemcitabine	8
Site of recurrence	
Lymph-nodes	24
Lung	9
Bone	15
Liver	3
Other	1
Subsequent therapies	
None	22
Paclitaxel	6

A total of 143 cycles were administered. The dose was reduced to 250 mg/m^2^ in 2 patients because of grade IV neutropenia. Overall dose intensity was 93.5%.

Toxicity is reported in Table 2. Grade III-IV neutropenia occurred in three patients (10.7%) despite granocyte-colony stimulating factors (G-CSF) prophylaxis. No neutropenic fever was reported. Grade III-IV anemia and Grade III thrombocytopenia were observed in 3 (10.8%) and 2 patients (7.2%), respectively. Four patients (14.2%) reported grade I-II constipation. Neither grade III-IV constipation nor other grade III-IV adverse events were observed. No patients discontinued treatment because of toxicity.

**Table 2:** Toxicity

	Grade I/II n (%)	Grade III n. (%)	Grade IV n (%)
Haematological			
Neutropenia	5 (17.8)	−	3 (10.8)
Neutropenic fever	−	−	−
Anemia	9 (32.2)	2 (7.1)	1 (3.6)
Thrombocitopenia	3 (10.7)	2 (7.2)	−
Non haematoligical			
Nausea	5 (17.8)	−	−
Dysgeusia	1 (3.6)	−	−
Constipation	4 (14.2)	−	−
Fatigue	5 (17.8)	−	−

**Figure 1:**
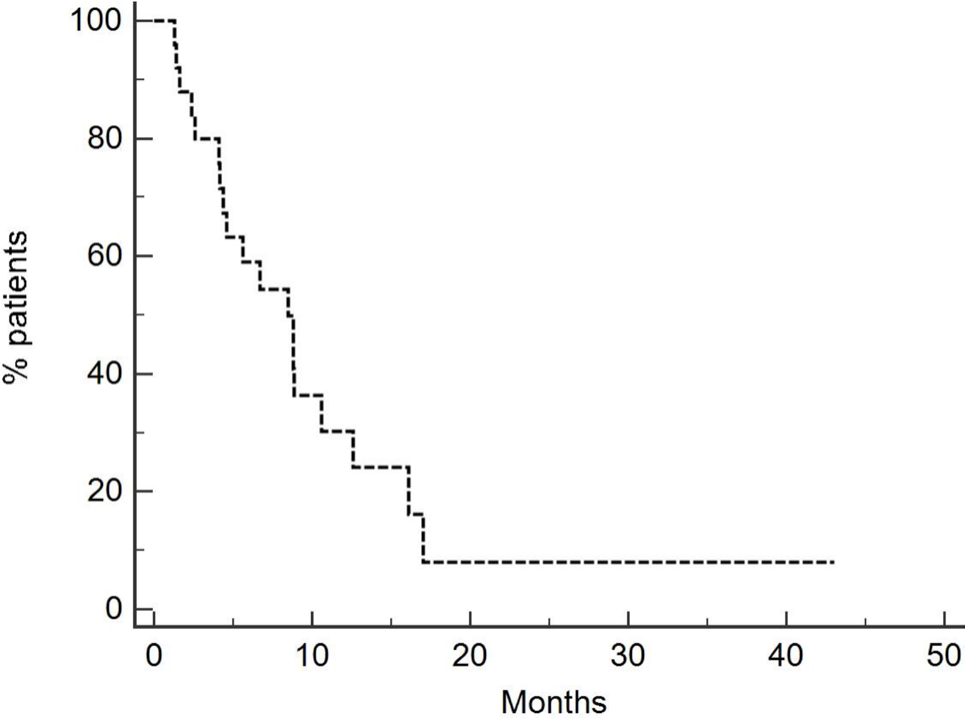
Overall survival of entire population (8.5 months) Twenty-eight patients were treated with Vinflunine at a dose of 280 mg/m^2^.

**Figure 2:**
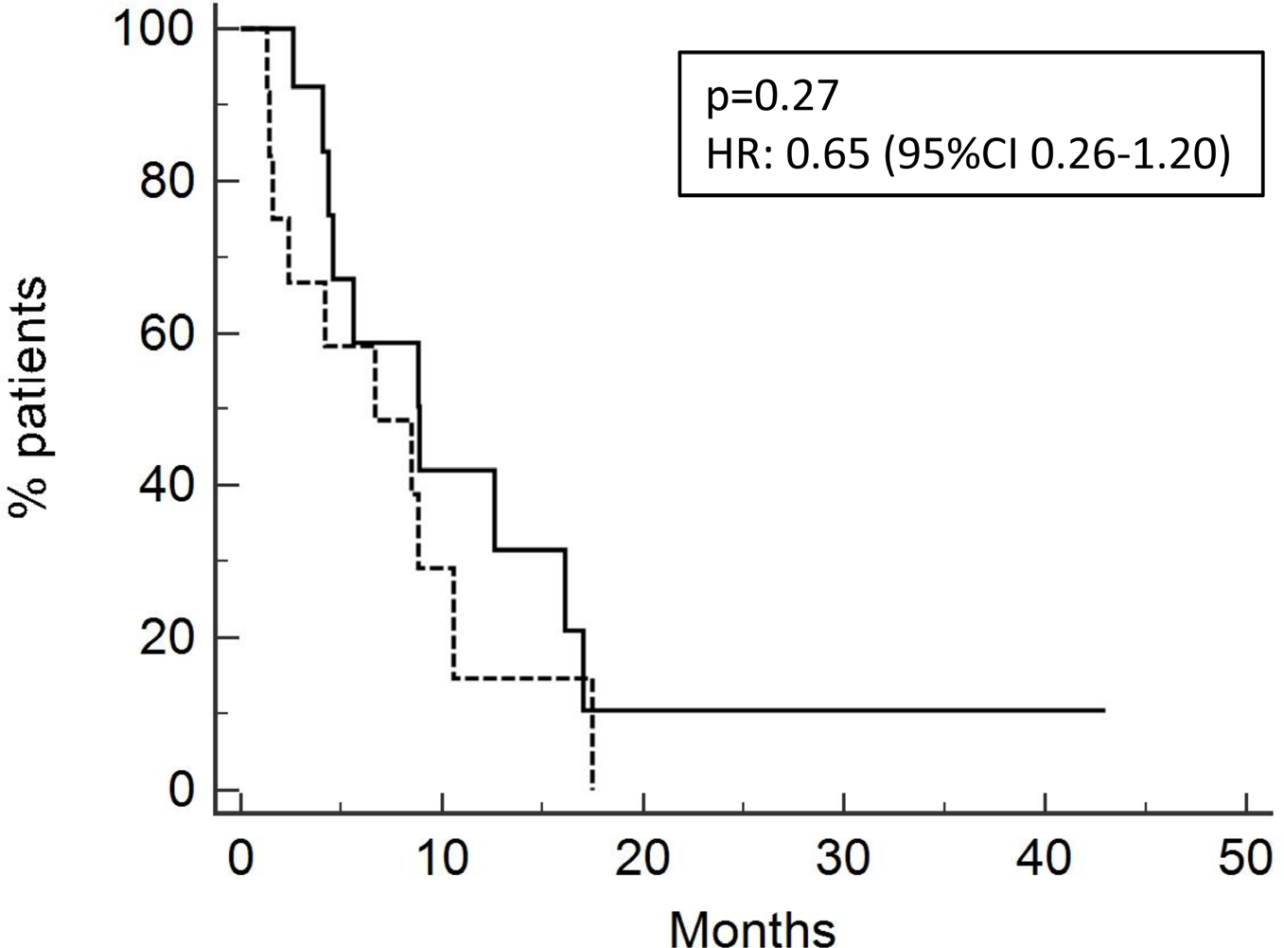
Overall survival of patients relapsed after neoadjuvant/adjuvant treatment (13 pts; OS 8.8 months, solid line) or after chemotherapy for metastatic disease (15 pts; OS 6.7 months, dotted line)

Overall survival was 8.5 months for the entire population (Figure 1), 8.8 months for patients treated with neoadjuvant/adjuvant chemotherapy and 6.7 months for patients previously treated with a first line chemotherapy (Figure 2) [*p* = 0.27, HR 0.65 (95-CI 0.26–1.20)].

**Figure 3:**
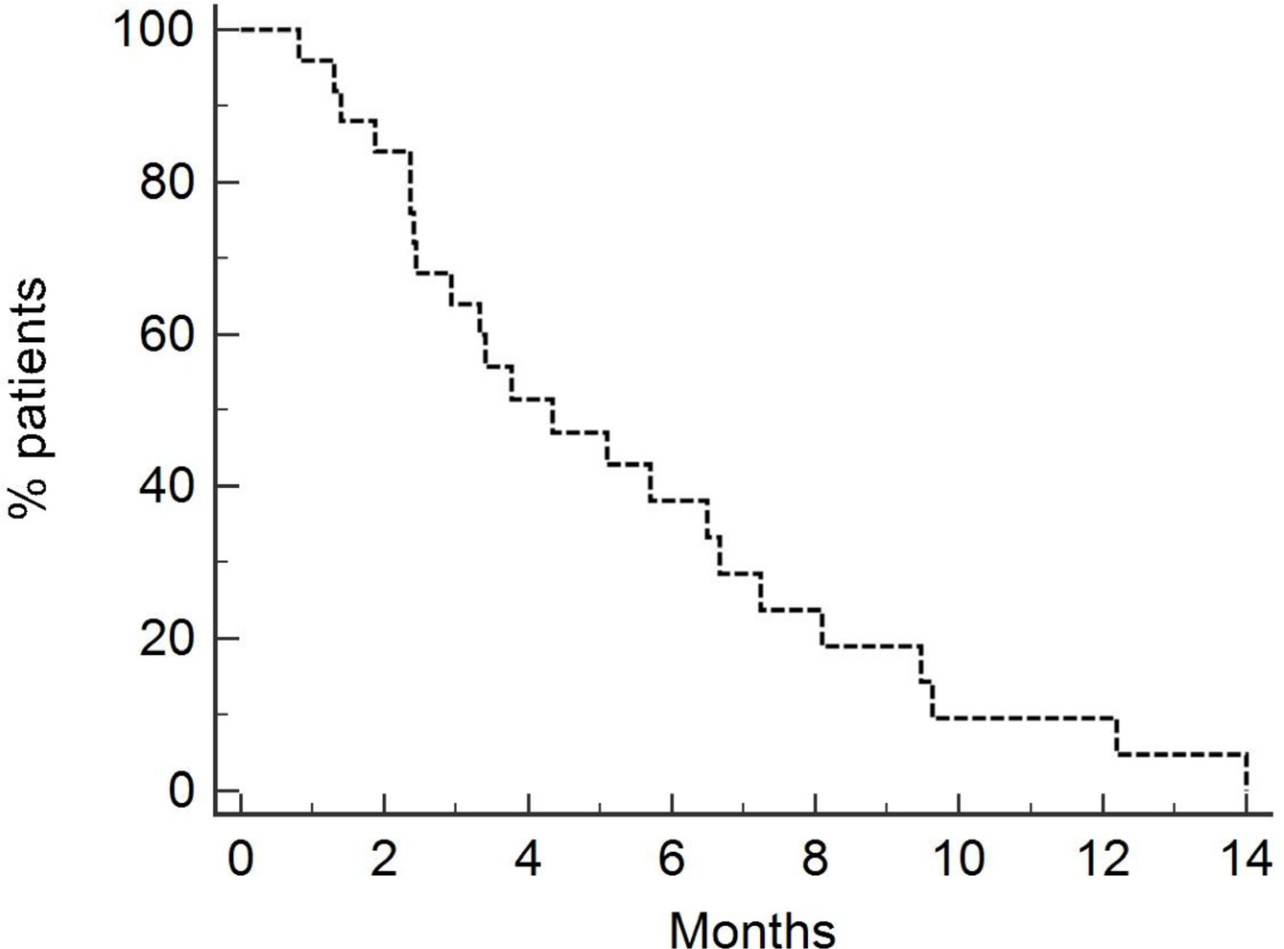
Progression-free survival of entire population (4.33 months) Twenty-eight patients were treated with Vinflunine at a dose of 280 mg/m^2^.

**Figure 4:**
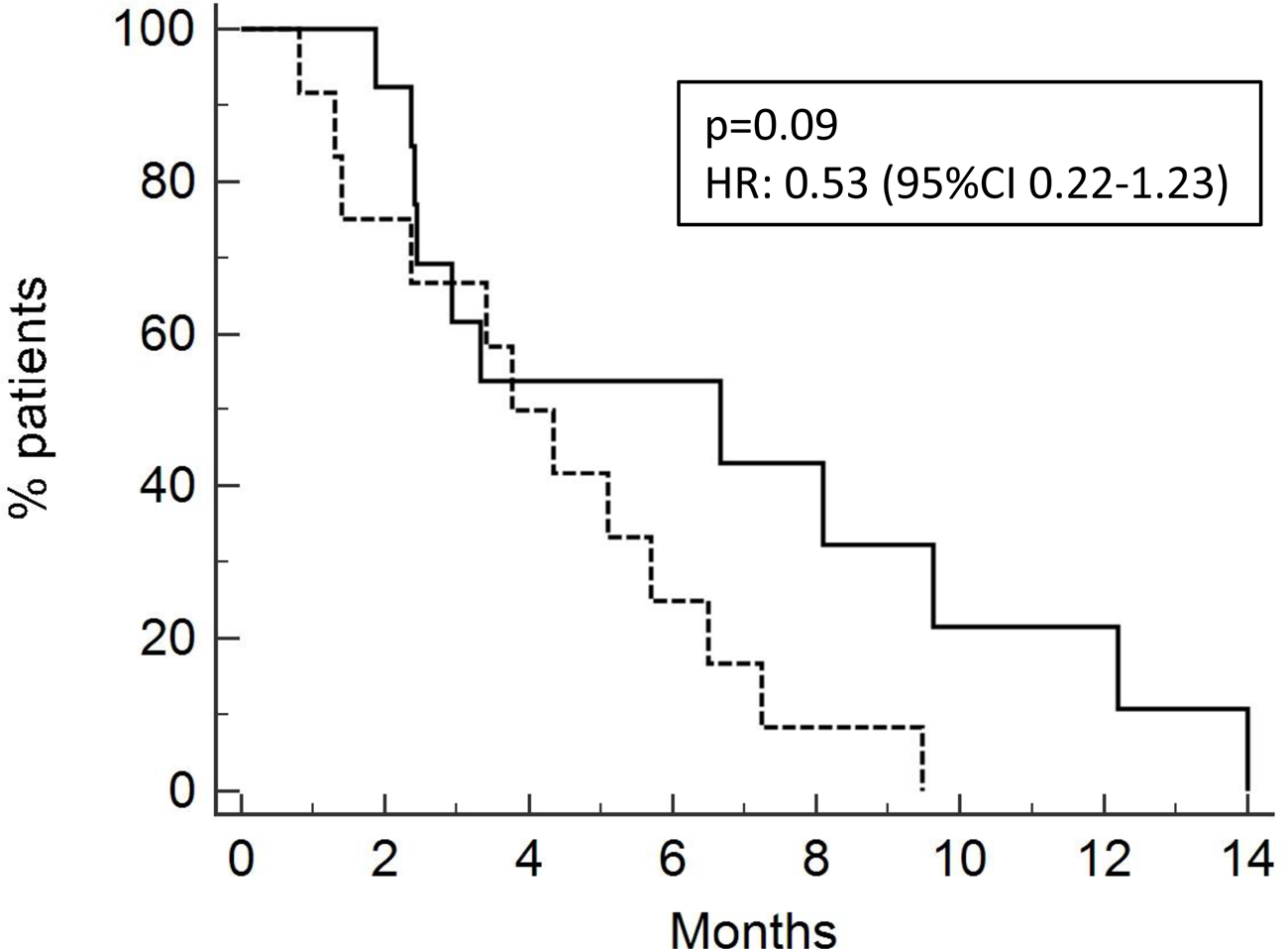
Progression-free survival of patients after neoadjuvant/adjuvant treatment (13 pts ; PFS 4.9 months, solid line) or after chemotherapy for metastatic disease (15 pts; 2.5 months, dotted line)

PFS for all patients was 4.33 months (Figure 3). PFS was 4.9 months and 2.5 months for patients who received neoadjuvant/adjuvant platinum + Gemcitabine and first line chemotherapy (Figure 4), respectively, [*p* = 0.09, HR 0.53 (95% CI 0.22–1.23)].

Responses are reported in Table 3. Complete response was observed in one patient (3.6%), partial response in 6 patients (21.4%) and stable disease in 9 patients (32.2%). Disease control was achieved in 16 patients (57.2%). Progression disease occurred in 12 patients (42.2). Seven patients (25%) underwent more than 8 cycles of therapy. The median response duration was 10.6 months.

**Table 3:** Response rate

	n. (%)
Complete response	1 (3.6)
Partial response	6 (21.4)
Stable disease	9 (32.2)
Disease control	16 (57.2)
Progression	12 (42.8)

## DISCUSSION

Effective agents for second line treatment of advanced TCCU are limited. Drugs commonly used in this setting could yield poor benefits. Due to the short survival, an acceptable quality of life should be achieved. Chemotherapy adverse events and frequent hospital admissions could negatively influence patients’ wellbeing.

Vinflunine is the only agent approved in Europe as second line therapy in advanced TCCU. In the phase III trial, survival advantages (6.9 months versus 4.3 months for BSC) were counterbalanced by a remarkable incidence of adverse events. In particular, 50% of the patients experienced grade III-IV neutropenia, 13.1% grade III-IV anemia, 16.1% grade III-IV constipation, 19.2% grade III-IV fatigue and 51.2% thrombocytopenia. The toxicity of Vinflunine limited its use, in addition to the restricted advantage on survival.

There is a lack of alternative drugs in this setting. In our study, we tested if the reduction of the dose associated with the use of prophylactic G-CSF and laxatives could allow effective results compared to a higher dose with a better toxicity profile. Indeed, we analyzed patients treated with the dose of 280 mg/m^2^ every 3 weeks after the failure of a previous platinum + Gemcitabine combination.

Overall survival observed in our study was 8.5 months, PFS 4.3 months, RR 25%, disease control rate 57.2%. These results are similar to those reported in the phase III trial and in other real life studies.

The remarkable duration of response (10.6 months) was obtained through the maintenance of dose intensity, to which the lower incidence of adverse events has contributed. The retrospective nature of the study could have influenced this result. The selection of patients with a high probability of prolonged response could be very useful in clinical practice. Unfortunately, in our study the identification of clinical predictive factors was not possible because of the limited number of patients.

The dose of 280 mg/m^2^, G-CSF as primary prophylaxis for neutropenia and laxative agents allow the reduction of the most common Vinflunine-related adverse events. As a matter of fact, grade III-IV neutropenia was 10.7%, instead of the rate of 50% reported in the phase III trial. Grade III-IV anemia and all grade thrombocytopenia occurred in 10.7% and 17.9%, respectively, compared to 19.1% and 51.2% reported by Bellmunt [19].

Concerning non haematological toxicity, in our population only grade I-II constipation and fatigue were observed (14.2% and 17.8%, respectively) whereas Bellmunt [19] described grade III-IV fatigue in 19.3% and grade III-IV constipation in 16.1%.

These data confirm a good tolerability of this drug and show a milder toxicity with the dose tested in our study. Prospective clinical trials should be helpful to confirm our observations. They could also represent a source for further pharmacokinetics information and for clinical and pathological predictive factors.

Despite being retrospective and including a limited number of patients, this study underlines that Vinflunine at a dose of 280 mg/m^2^ is effective and has a favorable toxicity profile. Vinflunine as administered in our study could be considered for the second line treatment of advanced TCCU also in the next future, when immunological checkpoint inhibitors [25] might be available in clinical practice.

## MATERIALS AND METHODS

Patients with advanced metastatic TCCU, PS (ECOG) 0–1, relapsed within 12 months after neoadjuvant/adjuvant platinum-based chemotherapy or progressed after a first line platinum-based treatment were included in this retrospective analysis. The dose of Vinflunine administered was 280 mg/m^2^ intravenously every 3 weeks for the entire population. Emollient laxatives were used for primary prophylaxis of constipation. Paraffin oil was prescribed fasting at the dose of 15 ml twice a day for 7 days after chemotherapy administration. The timing of administration was chosen based on the nadir of constipation, that occurs 3–5 days after Vinflunine administration. Granocyte-colony stimulating factors (G-CSF) were given as primary prophylaxis of neutropenia: Lenograstim 34 MU was administered for 6 days starting 24 hours after Vinflunine. We collected all demographic data related to patients: gender, age, previous treatments (in neoadjuvant/adjuvant or advanced setting), site of recurrence.

The primary outcomes were overall survival (OS) and toxicity; other outcomes were progression-free survival (PFS) and response rate (RR).

OS was calculated from the first day of cycle 1 to death and PFS from the first day of cycle 1 to progression or death. The Kaplan and Meier method was used; the comparison of survival between patients’ subgroups was obtained by the log-rank test. A Cox-proportional hazard model with 95% confidence interval (CI) was used to calculate the hazard ratios. All *p*-values were considered significant at the 5% level. Responses were evaluated trough RECIST criteria version 1.1.

Toxicity was reported using Common Terminology Criteria for Adverse Events (CTCAE) version 4. Data on tolerability were collected considering all the adverse events occurred throughout the treatment period. Clinical evaluations were performed every 3 weeks before Vinflunine administration. Visits were also performed monthly after the end of Vinflunine.

Our study was approved by local Ethic Committees and conducted according to the Helsinki Declaration. All the patients released a written informed consent.
